# DNA Vaccine Treatment in Dogs Experimentally Infected with *Trypanosoma cruzi*

**DOI:** 10.1155/2020/9794575

**Published:** 2020-05-05

**Authors:** Minerva Arce-Fonseca, Ana C. Carbajal-Hernández, Mónica Lozano-Camacho, Silvia del C. Carrillo-Sánchez, Francisco-Javier Roldán, Alberto Aranda-Fraustro, José Luis Rosales-Encina, Olivia Rodríguez-Morales

**Affiliations:** ^1^Department of Molecular Biology, Instituto Nacional de Cardiología “Ignacio Chávez”, Juan Badiano No. 1, Col. Sección XVI, Tlalpan, 14080 Mexico City, Mexico; ^2^Department of Echocardiography, Instituto Nacional de Cardiología “Ignacio Chávez”, Juan Badiano No. 1, Col. Sección XVI, Tlalpan, 14080 Mexico City, Mexico; ^3^Department of Pathology, Instituto Nacional de Cardiología “Ignacio Chávez”, Juan Badiano No. 1, Col. Sección XVI, Tlalpan, 14080 Mexico City, Mexico; ^4^Department of Infectomics and Molecular Pathogenesis, Centro de Investigación y de Estudios Avanzados-IPN, Av. Instituto Politécnico Nacional 2508, Col. San Pedro Zacatenco, Gustavo A. Madero, 07360 Mexico City, Mexico

## Abstract

Chagas disease is a chronic and potentially lethal disorder caused by the parasite *Trypanosoma cruzi*, and an effective treatment has not been developed for chronic Chagas disease. The objective of this study was to determine the effectiveness of a therapeutic DNA vaccine containing *T. cruzi* genes in dogs with experimentally induced Chagas disease through clinical, pathological, and immunological analyses. Infection of Beagle dogs with the H8 *T. cruzi* strain was performed intraperitoneally with 3500 metacyclic trypomastigotes/kg body weight. Two weeks after infection, plasmid DNA immunotherapy was administered thrice at 15-day intervals. The clinical (physical and cabinet studies), immunological (antibody and cytokine profiles and lymphoproliferation), and macro- and microscopic pathological findings were described. A significant increase in IgG and cell proliferation was recorded after immunotherapy, and the highest stimulation index (3.02) was observed in dogs treated with the pBCSSP4 plasmid. The second treatment with both plasmids induced an increase in IL-1, and the third treatment with the pBCSSP4 plasmid induced an increase in IL-6. The pBCSP plasmid had a good Th1 response regulated by high levels of IFN-gamma and TNF-alpha, whereas the combination of the two plasmids did not have a synergistic effect. Electrocardiographic studies registered lower abnormalities and the lowest number of individuals with abnormalities in each group treated with the therapeutic vaccine. Echocardiograms showed that the pBCSSP4 plasmid immunotherapy preserved cardiac structure and function to a greater extent and prevented cardiomegaly. The two plasmids alone controlled the infection moderately by a reduction in the inflammatory infiltrates in heart tissue. The immunotherapy was able to reduce the magnitude of cardiac lesions and modulate the cellular immune response; the pBCSP treatment showed a clear Th1 response; and pBCSSP4 induced a balanced Th1/Th2 immune response that prevented severe cardiac involvement. The pBCSSP4 plasmid had a better effect on most of the parameters evaluated in this study; therefore, this plasmid can be considered an optional treatment against Chagas disease in naturally infected dogs.

## 1. Introduction

Chagas disease or American trypanosomiasis is a zoonotic disease caused by the hemoflagellated protozoan *Trypanosoma cruzi*. This parasite is transmitted to domestic and wild mammals through the metacyclic trypomastigote-contaminated feces of hematophagous hemipteran insects belonging to the Reduviidae family, Triatominae (triatomines) subfamily, and it is known in Mexico as the “kissing bug.” Chagas disease is endemic to the American continent, and approximately 6-7 million people are estimated to suffer from American trypanosomiasis. More than 10,000 people die each year as a result of the disease, which has an annual incidence of 30,000 cases [1]. The cardiac form is the most serious and frequent manifestation of chronic Chagas disease, and it develops in 20%-30% of individuals and typically leads to conduction system abnormalities, bradyarrhythmias and tachyarrhythmias, apical aneurysms, cardiac failure, thromboembolism, and sudden death [2].

According to the World Health Organization classification, the endemic countries can be divided into four groups (I, II, III, and IV). Mexico fits within group II for complying with the following characteristics: intradomiciliary transmission evidence with a clear association is observed between *T. cruzi* infection and electrocardiographic alterations as well as other pathologies attributable to Chagas disease, and formal control programs have not been established [3].

Among the parasite reservoirs, the dog is considered the most important domestic species in the *T. cruzi* infection dynamics because dogs are an important source of food for triatomine insects, and they can also ingest infected bugs. Therefore, the risk of transmission within human dwellings by infected dogs has been proven [4]. Several studies have reported seroreactive dogs to the parasite in some regions of Mexico and an important seroprevalence in Morelos, Estado de México, Puebla, Yucatán, Chiapas, Campeche, Jalisco, Sonora, and Nuevo León [5, 6].

Chagas disease treatment involves two aspects: the symptomatic or nonspecific and trypanocide or specific. Chronic Chagas cardiomyopathy is still a challenging disease whose current and emerging treatment includes drugs, implantable cardioverter-defibrillators, permanent pacemakers, transcatheter ablation, heart transplantation, resynchronization therapy, and cell therapy focusing mostly on management of heart failure and arrhythmias [7, 8]. The use of drugs that eliminate the parasite is indicated for the treatment of acute symptomatic disease, which is acquired by vector, congenital, or accidental routes. On the other hand, the effectiveness of the trypanocidal treatment in chronic cases of the disease is controversial [9].

In the acute phase, it is necessary to administer the drug as quickly as possible and the dose is varied according to the patient's age and weight. Nifurtimox and benznidazole are the only two drugs with adequate trypanocide activity whose effect is against blood and tissue forms. The effectiveness of conventional chemotherapy is very low. In early infected children, treatment is successful in 55.8% of cases; however, in the chronic phase, most patients are resistant to therapy with conventional drugs and carry a lifelong infection [10, 11].

The usefulness of these drugs in patients with Chagas disease in the asymptomatic or symptomatic chronic phases has not been established. In addition, it has been reported that a large proportion of subjects treated with benznidazole experience severe side effects, including digestive manifestations and hematological, skin, and neurological alterations [11]. Experimental toxicity studies with both drugs evidenced neurotoxicity, testicular damage, ovarian toxicity, and deleterious effects in the adrenal, colon, esophageal, and mammary tissue as well as significant mutagenic effects [12]. Despite recent efforts to discover new treatments for Chagas disease, such as drug combinations, drug repositioning, redosing schemes for current drugs, and identifying new drugs with specified target profiles or additive or synergistic interactions of compounds with different modes of actions, better safety and greater effectiveness of drug treatment is not yet available [13–15].

DNA vaccines are currently under research, and their potential use for both the prevention and the treatment of a variety of infectious diseases has been explored, including for Chagas disease [16–20]. The vast majority of our knowledge about immune mechanisms and protective response for *T. cruzi* infection comes from experimental animal models [21]. The use of a DNA vaccine as a treatment in Chagas disease has been partially successful in animal models as demonstrated by Dumonteil et al., who infected mice with *T. cruzi* and then treated them with DNA coding for parasite antigens and found less parasitemia, reduced cardiac tissue inflammation, and increased survival [20]. One of the most important advantages of using canine models in relation to other animal models is the advanced knowledge and similarity of the cardiac morphology and physiology of the heart conduction system with humans. Several clinical aspects of the disease similar to those verified in humans have been observed in dogs, thus leading to the possibility of performing electrocardiographic monitoring of the infected animal and verifying the correlations between these alterations and cardiac conduction system lesions and offering good interpretation of the results [21].

Vaccines against Chagas disease have been previously tested in dogs by our group [22–24] and by others [25–27]. The results, which focus mainly on the immune response and cardiac damage evaluated by histology, have been variable, although in all cases, the infection is not avoided and the degree of protection ranges from mild to moderate.

The aim of our study is to evaluate the therapeutic efficacy of the administration of plasmid DNA coding for the TcSPP4 and TcSP antigens of *T. cruzi* in dogs during the acute and chronic phases of Chagas disease through clinical, pathological, and immunological analyses of Beagle dogs infected with the H8 *T. cruzi* autochthonous Mexican strain.

## 2. Materials and Methods

### 2.1. Experimental Animals

**Figure 1:**
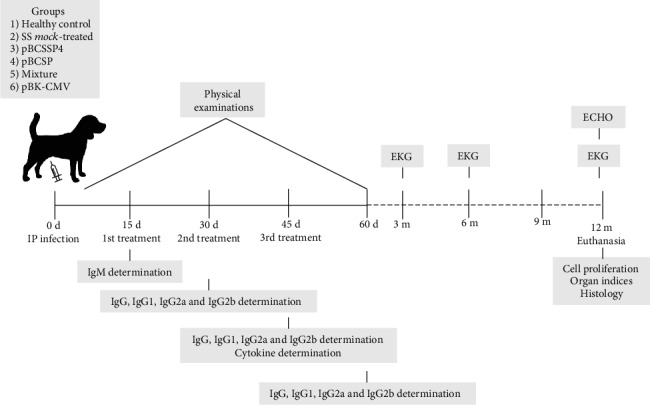
Schematic representation of the methodological design. Five groups of dogs were intraperitoneally infected with 3500 metacyclic trypomastigotes/kg body weight of the H8 *T. cruzi* strain to evaluate the effectiveness of the therapeutic DNA vaccine containing *T. cruzi* genes.

Thirty male and female four-month-old Beagle puppies from healthy parents were subjected to a basic calendar of preventive medicine that included vaccination and deworming. All animals were tested for the absence of antibodies against *T. cruzi* using the enzyme-linked immunosorbent assay (ELISA). The dogs were separated in six experimental groups, which are detailed in Table 1. The healthy group included noninfected/untreated dogs as the control; the SS *mock*-treated group was used as the positive control of infection; the pBCSSP4 group included infected dogs treated with the pBCSSP4 plasmid; the pBCSP group included infected dogs treated with the pBCSP plasmid; the mixture group included infected dogs treated with the pBCSSP4 and pBCSP plasmids; and the pBK-CMV group included infected dogs treated with the empty cloning vector plasmid. A representation of the experimental design is shown in Figure 1.

Animal handling followed the established guidelines of the International Guiding Principles for Biomedical Research involving Animals and the Norma Oficial Mexicana (NOM-062-ZOO 1999) Technical Specifications for the Care and Use of Laboratory Animals [28], and the experimental protocol was approved by the Research and Bioethics Committees of the Instituto Nacional de Cardiología, Ignacio Chávez (Registration number: 11-737).

### 2.2. Blood Samples

Blood extraction was performed directly from the cephalic vein. The dorsal area of the foreleg was constricted at the level of the elbow to raise the vein, and the puncture was started above the metacarpal joint using disposable 3 mL syringes with 21 G × 32 mm needles (PROTEC, Mexico). The sample was collected and immediately deposited in tubes without anticoagulant. Subsequently, the clot was removed and the serum was separated by centrifugation at 3500 rpm for 15 min. Sera were frozen at -20°C until use.

### 2.3. Diagnostic Serology for Chagas Disease

Before infecting the dogs, it was determined that they were not naturally infected with the parasite. After experimental infection, an enzyme-linked immunosorbent assay (ELISA) and indirect immunofluorescence (IIF) as a confirmatory test were used to determine IgM and IgG antibodies against *T. cruzi* as previously described [5, 29].

### 2.4. Physical Examinations

Physiological constants, inspections, auscultation, palpation, and percussion were performed during the general physical examinations in all animals. Parameters such as body weight, rectal temperature, body condition, mental state, heart rate, respiratory rate, heart auscultation, lung fields, lung field palm percussion, arterial pulse, mucous membranes, capillary refill time, lymph node palpation, dehydration percentage, head and face natural orifice examinations, cough reflex, swallowing reflex, and abdominal palpation were evaluated and registered [30].

### 2.5. Infection

Dogs were infected by intraperitoneal injection of 3500 metacyclic trypomastigotes of the H8 *T. cruzi* strain (MHOM/MX/1992/H8 Yucatán (*T. cruzi*)) [31]/kg body weight. The parasites were obtained from the urine and feces of triatomes and resuspended in saline solution (SS).

### 2.6. Plasmid Descriptions

Plasmids based on the pBK-CMV commercial plasmid vector (Stratagene (now Agilent Technologies), CA, USA) encoding the *T. cruzi* antigens TcSP and TcSSP4 have been described previously [22, 32, 33]. Briefly, pBK-CMV has 17 cloning sites flanked by the T3 and T7 promoters and contains the cytomegalovirus early promoter, which allows for eukaryotic expression and the polyadenylation sequence of the SV40 virus, thus providing the signal required for the termination of eukaryotic transcription and polyadenylation. This plasmid has a kanamycin resistance gene that allows for the selection of positive clones in bacteria. This plasmid was used for the construction of those carrying the *T. cruzi* genes, which will be used for immunotherapy against Chagas disease. pBK-CMV was also used as an empty vector control for the DNA treatment. The pBCSP plasmid is a construct derived from the pBK-CMV vector that possesses the gene coding for the TcSP protein of *T. cruzi*, an all stages-expressed *trans*-sialidase that adds sialic acid to the mucins of the surface cover for host-parasite interactions. pBCSSP4 is a construct derived from the pBK-CMV vector that possesses the gene coding for the TcSSP4 protein of *T. cruzi*, an acid glycoprotein that is expressed during the transformation of trypomastigotes into amastigotes.

### 2.7. Plasmid DNA Purification

Under aseptic conditions, the transformed *Escherichia coli* XL1-Blue strain carrying the pBCSSP4, pBCSP, and pBK-CMV plasmids was cultured in 500 mL of Luria Bertani broth with kanamycin by 16 h at 37°C/200 rpm incubation. Plasmid DNA was purified by alkaline lysis and ultrapurified using Qiagen (Hilden, Germany) columns. DNA used for immunotherapy was resuspended in lipopolysaccharide-free PBS (Gibco by Thermo Fisher, MA, USA), and its purity was estimated and quantified [34]. Aliquots of 0.5 mg dissolved in 0.5 mL of SS were generated and stored at -20°C until use.

### 2.8. Plasmid DNA Immunotherapy

At day 15 postinfection, the dogs were treated thrice at 15-day intervals by intramuscular injection of 500 *μ*g of each recombinant plasmid (pBCSP or pBCSSP4) or a combination of 250 *μ*g of each plasmid or vector DNA (pBK-CMV) in the semitendinosus and semimembranosus muscles of the pelvic members. The SS *mock*-treated control animals were injected with 500 *μ*L of sterile SS on the same schedule as the treated dogs.

### 2.9. Antibodies Determination

Total IgM and IgG immunoglobulins as well as the IgG1, IgG2a, and IgG2b isotypes were evaluated 15 days postinfection (IgM) and 15 days after each treatment (total IgG, IgG1, IgG2a, and IgG2b) by the ELISA method using a whole protein extract of the *T. cruzi* INC-9 isolate as the antigen as described previously [5, 29]. Briefly, 96 MaxiSorp plates (Nunc by Thermo Fisher, MA, USA) were coated with the whole *T. cruzi* isolate extract (1 *μ*g/mL) overnight at 4°C in 200 *μ*L of NaCO_3_/NaHCO_3_ pH 9.6 (carbonate buffer). The plates were washed seven times with 215 *μ*L PBS 1X-0.05% Tween-20 (PBS-T) and blocked with 200 *μ*L 0.5% BSA in PBS-T (blocking buffer) for at least 30 min at 37°C. Serum samples were diluted in blocking buffer at a dilution of 1 : 200 in 200 *μ*L/well and incubated (1 h, 37°C). Plates were then washed seven times, and 200 *μ*L of peroxidase-conjugated anti-dog immunoglobulin G (IgG), IgG isotypes (IgG1, IgG2a, and IgG2b), or immunoglobulin M (IgM) secondary antibodies (Novus Biologicals, CO, USA) was added and incubated (1 h, 37°C). The conjugates were diluted in blocking buffer at 1 : 10,000. The plates were washed seven times, and 150 *μ*L of peroxidase substrate OPD (*ortho*-phenylenediamine dihydrochloride, Sigma-Aldrich, MO, USA) in citrate buffer at pH 4.5-0.03% H_2_O_2_ was added. The reaction was stopped 10 min later by the addition of 50 *μ*L of 5 N H_2_SO_4_. Absorbance values were determined at 495 nm in a Microplate Reader (Bio-Rad, CA, USA). All measurements were performed twice, and the data presented are the mean of the values for each dog.

### 2.10. Cytokine Quantification

The IL-1 alpha, IL-6, IL-12, IFN-gamma, and TNF-alpha levels in the sera of immune-treated dogs at 3, 8, 12, and 24 h after the last treatment dose were measured by ELISA using commercial kits (PeproTech, NJ, USA) in accordance with the manufacturer's instructions as follows. The capture antibody was diluted to 1.0 *μ*g/mL with PBS, and 100 *μ*L/well was added to the 96 MaxiSorp plates (Nunc) and incubated overnight at room temperature. The plates were washed four times with 215 *μ*L PBS-T, blocked with 200 *μ*L of blocking buffer per well for 1 h at room temperature, and washed four times. The standard sample was prepared for each cytokine, and 100 *μ*L/well was added; then, 100 *μ*L of the immune-treated dog serum was added to each well and incubated 2 h at room temperature. The plates were washed four times, and then, 100 *μ*L of the previously diluted detection antibody was added and incubated at room temperature for 1.5 h. The plates were washed, and then, 100 *μ*L of previously diluted avidin was added and incubated 30 min at room temperature. The plates were washed again, and then, 100 *μ*L of peroxidase substrate OPD (Sigma-Aldrich) in citrate buffer at pH 4.5-0.03% H_2_O_2_ was added. The plates were incubated 15 min at room temperature, and the reaction was stopped with 50 *μ*L of 5 N H_2_SO_4_ per well. The reading was performed at 490 nm using a Microplate Reader (Bio-Rad, Model 550).

### 2.11. Cell Proliferation

At 10.5 months after the last treatment with the plasmid DNA (12 months postinfection), the proliferative response of spleen cells was studied *in vitro*. Splenocytes were obtained by necropsy, washed three times in Hank's solution (Sigma-Aldrich), and resuspended in Dulbecco's Modified Eagle Medium (DMEM, Gibco) supplemented with 1 mM nonessential amino acids, 10% fetal bovine serum, 2 mM L-glutamine, and 50 *μ*M beta-mercaptoethanol at a concentration of 4 × 10^5^ cells/mL. The viability percentage was obtained by exclusion with Trypan blue staining. The cells were cultured in 96-well flat bottom plates (Corning, NY, USA); and then, 100 *μ*L of cell suspension and 10 *μ*g/mL antigen (whole protein extract of epimastigotes of *T. cruzi* INC-9 isolate) were added into each well. Concanavalin A (Sigma-Aldrich) was added at a concentration of 5 and 10 *μ*g/mL as a positive control. Each determination was performed in triplicate. The plates were incubated at 37°C in a 5% CO_2_ for 120 h (or 72 h for Concanavalin A). At 16 h prior to the end of the incubation, 0.5 *μ*Ci of [^3^H]-thymidine (Amersham, Buckinghamshire, UK) was added to each well. The lymphocytes were collected with a manual cell harvester (Nunc), and the amount of incorporated radioactive thymidine was measured using liquid scintillation spectroscopy (Beckman Coulter, CA, USA, model LS 5801). The stimulation of a specific cellular immune response is represented by the stimulation index (S.I.) by the average of the data obtained in triplicate and estimated as follows: mean counts per minute of stimulated cultures/mean counts per minute of nonstimulated cultures. S.I. values above 2.5 were considered positive [35].

### 2.12. Electrocardiography

To determine whether the treatment with recombinant DNA plasmids has any effect on the electrical conduction of the heart during the chronic stage of the disease, electrocardiographic recordings were performed for all animals at three, six, and 12 months after infection. The dogs were held by an attendant in right lateral recumbency, and no chemical restraint was employed; such manipulation was achieved via previous training through daily manipulation on examination tables to maintain the dogs at the desired position to carry out the study. Peripheral bipolar standard leads (I, II, and III), augmented unipolar peripheral leads (aVR, aVL, and aVF), and special leads (unipolar precordial thoracic leads: CV_5_RL, CV_6_LL, CV_6_LU, and V_10_) were recorded (Schiller, FL, USA). For each tracing, the voltage was standardized at 1 mV/cm and the paper speed was 50 mm/s.

### 2.13. Echocardiography

Transthoracic echocardiography (Philips, Amsterdam, Netherlands, model IE33) with a 2-5-3.5 MHz probe was performed in all dogs during the chronic stage of infection (12 months after inoculation) to detect and compare morphological changes in the dogs' hearts. Most of the animals were positioned in dorsal and right or left lateral decubitus without any chemical restriction during the study, which was achieved via previous training through daily manipulation on examination tables to maintain the dogs in the desired position to carry out the study. Image acquisition was performed via a long parasternal axis and two- and four-chamber apical view in bidimensional mode. The end-diastolic and end-systolic diameters of the left ventricle were measured. The left ventricle (LV) septum walls, posterior wall, left atrium (LA), and aorta root (AR) were also recorded. The parameters of LV systolic function, i.e., fractional shortening (FS %), and left ventricular ejection fraction (LVEF %) were calculated from the end-diastolic and end-systolic volumes in the standard four-chamber long-axis two-dimension echo views and following the formula [end‐diastolic volume–end‐systolic volume]/end‐diastolic volume × 100. The LA size parameter was calculated from the ratio of LA and AR. The averages and standard deviations of all parameters were obtained for each group.

### 2.14. Euthanasia and Organ Indices

At 12 months postinfection, chronic chagasic dogs were euthanized according to the Norma Oficial Mexicana (NOM-033-SAG/ZOO-2014) Methods to Bring Death upon Domestic and Wild Animals [36] by direct intravenous injection of sodium pentobarbital (Barbithal, Holland Animal Health, Mexico) at doses of 150 mg/kg into the cephalic vein. Prior to euthanasia, the animal weight was obtained (Bascule Inpros SA de CV, Mexico), and the heart, the spleen, and the popliteal lymph nodes were collected during necropsy and were weighed. Cardiomegaly, splenomegaly, and lymphadenopathy were evaluated by inspecting macroscopic alterations and determining the heart, spleen, and lymph node indices (organ weight/total body weight × 100), respectively. The presence of cardiomegaly and splenomegaly was considered when the organ index was significantly higher than that observed in the organs from healthy noninfected animals [23, 37].

### 2.15. Histology

Longitudinal and transversal right ventricle (RV) and LV heart muscle tissues were obtained. Tissue sections were fixed in 10% buffered formalin for 24 h. Samples were dehydrated in absolute ethanol, rinsed in xylene, and embedded in paraffin. Noncontiguous sections at 5 *μ*m thickness were cut and stained with hematoxylin and eosin and evaluated by light microscopy (Carl Zeiss, K7, Germany). Images were obtained through a BioDoc-It Imaging System image analyzer (UVP, LLC, USA). At least 20 random microscopic fields (100 and 400x) were analyzed in each microscopic section using the open-source ImageJ software (NIH, USA). The severity of inflammation in the affected tissue was scored on a scale of 1 to 4. A score of 1 indicated one or less foci of inflammatory cells/field (400x); 2 indicated more than one focus of inflammatory cells/field; 3 indicated generalized coalescing foci of inflammation or disseminated inflammation with cell necrosis and retention of tissue integrity; and 4 indicated diffuse inflammation, tissue necrosis, interstitial edema, hemorrhage, and loss of tissue integrity.

### 2.16. Statistical Analysis

Continuous variables, such as body temperature, heart rate, antibody, or cytokine presence, and echocardiographic parameters were analyzed using the one-way or two-way ANOVA statistical test (SPSS software, version 17.0) followed by Tukey's analysis establishing a correlation between each experimental group and the control one. Nonparametric data, such as lymph node palpation, heart and spleen indices, and histological data, were analyzed by the Kruskal-Wallis test (SPSS software, version 17.0). In all cases, differences were considered significant at *P* < 0.05.

## 3. Results

### 3.1. Immunotherapy with Recombinant Plasmids Containing *T. cruzi* Genes Controlled Some Signs of Chagas Disease in Infected Dogs

The data from the physical examination were under the reference values or showed that all the dogs were healthy before the start of the project. The acute phase presentation of Chagas disease was characterized by fever, swelling of lymph nodes, pale mucous membranes, slow capillary refill time, anorexia, and slight weight loss, and it was observed in 100% of the infected/nontreated dogs from days 5 to 35-50 postinfection; however, in the immunotreated groups, only 25% of each group showed a mild fever and lymph node inflammation from days 22 to 30 postinfection (days 7 to 15 posttreatment).

### 3.2. *T. cruzi* Infection in Beagle Dogs Was Confirmed with Serological Diagnostic Tests

The ELISA serological test showed that all of the experimental animals were negative for the diagnosis of Chagas disease, which confirmed that the dogs were free of *T. cruzi* infection before any manipulation. Before the first immunotherapy, *T. cruzi* infection was demonstrated at day 15 postinfection by the detection of specific IgM anti-*T. cruzi* antibodies in all dogs (data not shown) and at 30 days postinfection by the detection of specific IgG anti-*T. cruzi* antibodies in all infected/nontreated groups by the ELISA method and IIF confirmatory test.

### 3.3. Immunotherapy Was Not Able to Differentially Modulate the Humoral Immune Response in Treated or Untreated Dogs Experimentally Infected by *T. cruzi*

**Figure 2:**
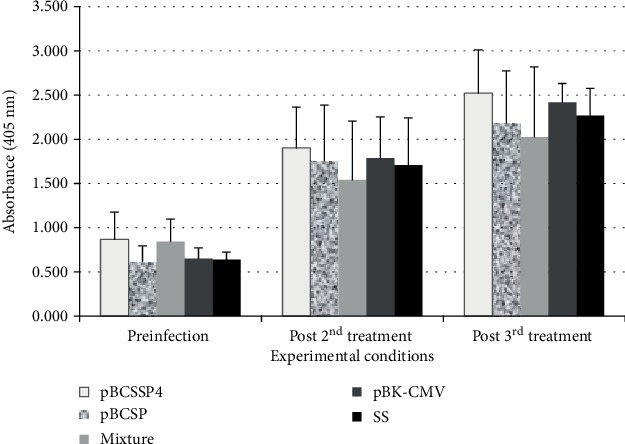
Total IgG titers in *T. cruzi*-infected dogs treated with plasmid DNA. ELISA was performed at different times to evaluate the serum levels (absorbance in optical density at 405 nm) of *T. cruzi* IgG-specific antibodies. Preinfection; post 2^nd^ treatment: 45 days after infection and 15 days after the second immunotherapy; and post 3^rd^ treatment: 60 days after infection and 15 days after the third immunotherapy. The values represent the average of triplicate assays ± S.D. Significant differences (^∗^*P* ≤ 0.05) were not identified.

To determine the effect of the therapeutic DNA vaccine on specific humoral responses against *T. cruzi* infection in chagasic dogs, antibodies were detected by ELISA. A significant increase in IgG was generated after 45 and 60 days postinfection in all groups (Figure 2) regardless of whether the dogs were vaccinated or not. In addition, a Th1- or Th2-polarized immune response was not observed after immunotherapy or infection based on the detection of IgG2a and IgG2b or IgG1, respectively; in other words, both subclasses remained at similar levels and there was no difference between them (data not shown).

### 3.4. pBCSSP4 Plasmid Was Able to Trigger a Balanced Response (Th1/Th2) by High IL-1 and IL-6 Production, While pBCSP Induced a Th1 Immune Response Profile by High IFN-Gamma, TNF-Alpha, and IL-1 Levels

**Figure 3:**
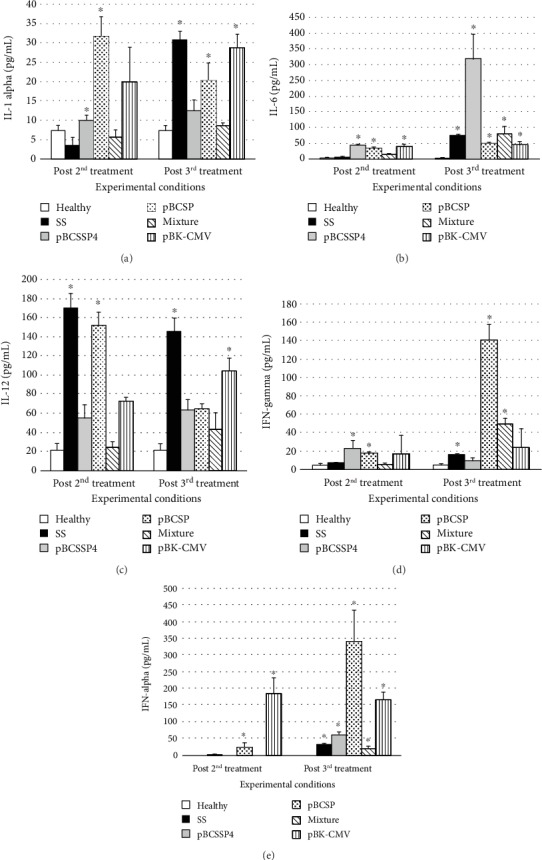
Serum level of cytokines in dogs infected with *T. cruzi* and treated with DNA vaccines. An ELISA was performed at 3 h after each treatment to evaluate the serum levels (absorbance in optical density at 405 nm) of each cytokine in dogs. (a) IL-1 alpha, (b) IL-6, (c) IL-12, (d) IFN-gamma, and (e) TNF-alpha. Post 2^nd^ treatment: 30 days after infection; post 3^rd^ treatment: 45 days after infection. The values represent the average of triplicate assays ± S.D. (^∗^*P* ≤ 0.05).

To evaluate the immunotherapy with the DNA vaccine on specific cellular immune responses against *T. cruzi* infection, the IL-1 alpha, IL-6, IL-12, IFN-gamma, and TNF-alpha cytokine levels were determined. Kinetics of the serum levels of these cytokines was quantified at 3, 8, 12, and 24 h after each treatment. The optimal time for all cytokines was at 3 h posttreatment because there were detectable levels of all cytokines at this time; therefore, only the analysis of the amounts of each cytokine obtained during the 3 h posttreatment is shown (Figure 3). According to the expected effect of the infection on cytokine production, it was possible to demonstrate that the infection itself induces a significant increase in the production of IL-12 in these dogs after 30 days of infection without treatment in comparison with the control group of healthy dogs (Figure 3(c)). As the infection progressed (at day 45 postinfection), the differences between these both groups were significant in all cytokines (Figure 3).

#### 3.4.1. IL-1 Alpha

The separate administration of recombinant plasmids significantly stimulated the production of IL-1 alpha (Figure 3(a)) after the second treatment dose, and it was better than that of the pBCSP plasmid with an increase of approximately 10-fold. After the administration of the third treatment dose, the production of this cytokine was significantly increased by the pBCSP and pBK-CMV plasmids (Figure 3(a)) without exceeding the stimulation by the infection alone. None of the other treatments had a significant effect on stimulating the production of this cytokine at either time.

#### 3.4.2. IL-6

The recombinant plasmids and the empty vector stimulated the production of IL-6 (Figure 3(b)) after the second dose of treatment. The mixture of both plasmids and the infection alone had no effect. With the third treatment (45 days after infection), the production of IL-6 in the group treated with *TcSSP4* gene was significantly increased by 4-fold above the SS *mock*-treated infected group while the *TcSP* gene, the mixture of both plasmids, and the pBK-CMV empty plasmid induced similar levels of this cytokine to those of the infected/SS *mock*-treated group (Figure 3(b)).

#### 3.4.3. IL-12

The serum level of IL-12 (Figure 3(c)) increased with the second treatment of pBCSP plasmid and was as high as the infection alone, while the pBCSSP4 plasmid, the mixture of recombinant plasmids, and the empty vector did not have a significant effect. The empty vector showed similar values to those of the infected/SS *mock*-treated group in the third treatment, while the two genes separately and the mixture of genes had levels similar to those of the healthy control group, demonstrating that they did not stimulate IL-12 production any more than the infection alone. In fact, the levels were lower than those of the infected/SS *mock*-treated group.

#### 3.4.4. IFN-Gamma

Significant IFN-gamma production (Figure 3(d)) was induced by both recombinant plasmids separately. The mixture of both plasmids and the empty vector and the infection alone had no effect at this time. With the third treatment, the pBCSP plasmid increased the levels of this cytokine by approximately 10-fold compared with those of the infected/SS *mock*-treated dogs. The mixture of both plasmids also had a positive effect after the third treatment, although at a smaller proportion.

#### 3.4.5. TNF-Alpha

After the second treatment, only the empty vector and the pBCSP plasmids increased the production of TNF-alpha (Figure 3(e)). With the third treatment, a significant increase was recorded in all groups compared with the control healthy group; however, those treated with the pBCSP plasmid had a 14-fold higher level of this cytokine than the infected/SS *mock*-treated group.

To summarize, immunotherapy with the pBCSSP4 plasmid was able to trigger a balanced response (Th1/Th2) by high IL-1 and IL-6 production while the treatment with the pBCSP plasmid induced a Th1 immune response profile based on the high IFN-gamma, TNF-alpha, and IL-1 levels.

### 3.5. Cell Proliferation Was Mostly Stimulated by the pBCSSP4 Recombinant Plasmid than by pBCSP

**Figure 4:**
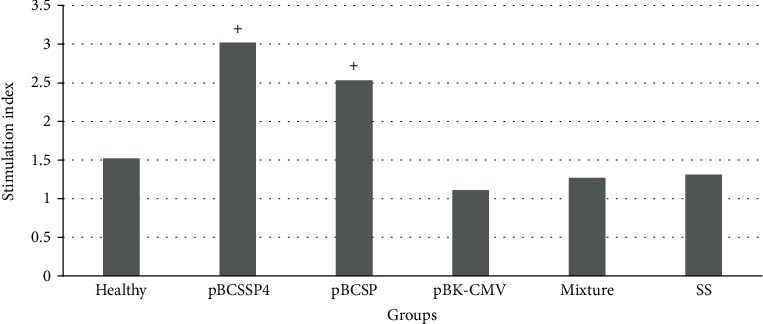
Lymphoproliferation of spleen cells of *T. cruzi*-infected dogs treated with plasmid DNA. The values represent the stimulation index and were considered positive (+) if they were equal to or above 2.5 [35].

To evaluate the specific cellular immune response in infected dogs treated intramuscularly with *T. cruzi* genes, the lymphoproliferative response to stimulation with parasite antigens was studied. The lymphoproliferative responses were observed in the animals treated with both *T. cruzi* genes separately (Figure 4). The highest stimulation index (3.02) was observed in dogs treated with the pBCSSP4 plasmid, and proliferation of the splenocytes was also observed in the dogs that received the immunotherapy with the pBCSP plasmid (stimulation index 2.53). Proliferation occurred when the stimulation index is above 2.5 [35]. In the control groups and group treated with the mixture of the two recombinant plasmids, cell proliferation was not observed and the stimulation index values were below 2.5. The cultures stimulated with Con A showed a value of 8.65 ± 0.79, thus demonstrating the viability of the cells in all experimental groups.

### 3.6. Electrocardiograms (EKG) of Dogs Immunotreated with Recombinant Plasmids Showed Few Nonserious Abnormalities

The DNA vaccines used as immunotherapy in the dogs infected with *T. cruzi* were moderately effective in preventing cardiac disturbances or delaying the onset associated with chronic chagasic cardiomyopathy evaluated by electrocardiography at three, six, and 12 months after infection (Table 2). In all groups, sinus arrhythmia was found at least once in the twelve months, and this condition was likely due to a physiological state associated with the physical restraint procedures related to respiration. The group treated with either the recombinant plasmids or the mixture had cardiac disturbances limited to only one alteration or in combination with two other abnormalities that did not represent a serious pathology at three months postinfection. In contrast, those animals that received treatment with pBK-CMV or SS showed a combination of four alterations, such as the mean electrical axis deviation (MEAD) of less than +40°, QRS complex with a wide R wave, the absence of T waves in some recordings, and S-T segment elevation of 0.5 mV in II, III, aVF, and CV_6_LL in 60% of the animals. At six months postinfection, electrocardiographic abnormalities were only registered in 20% and 40% of the dogs in the groups treated with pBCSP and pBCSSP4, respectively. The EKG with the greatest number of abnormalities occurred in 80% of the individuals (4/5) in the empty plasmid or infected/SS *mock*-treated groups. By 10 months postinfection, an individual from the pBK-CMV group experienced sudden death; and in its last EKG, this dog had presented MEAD to the right; right bundle branch block (RBBB); ventricle enlargement, infarction, and ischemia; areas of myocardial infarction; adhesions between pericardium and pleura; and apical aneurysm of approximately 0.5 cm at necropsy. Finally, at 12 months postinfection, arrhythmia in combination with MEAD to the left, ventricle enlargement, ischemia, and microscopic intramural myocardial infarction were found in the pBK-CMV group, whereas both arrhythmia and ischemia in combination with MEAD to the left and/or to the right and ventricle enlargement were found in the SS *mock*-treated group.

### 3.7. Therapeutic DNA Vaccine with pBCSSP4 Showed a Better Protective Effect on Cardiac Function by Echocardiography

Each average value was compared with the reference values and the parameters from the dogs of the healthy group (Table 3). The diastolic and systolic diameters of the groups that received immunotherapy were not affected since the values were very similar to those of the healthy group, whereas significantly lower values were observed in the infected/SS *mock*-treated group. The shortening fraction of the LV was not affected by the infection or modified with immunotherapy, which indicates an efficient contractile force in all groups. The thickness values of the posterior wall and the septum in the dogs' hearts did not show significant differences with respect to the healthy group and were within the reference values except for the group treated with pBCSSP4, whose values resembled those of the infected/SS *mock*-treated group, suggesting hypertrophic cardiomyopathy. The pBCSP group had significantly lower LV ejection fraction values (43%) than the rest of the groups (49%-50%) and the healthy group (53%), which indicates significant myocardial injury probably due dilated heart disease caused by *T. cruzi* infection. This finding is supported by the ratio of the left atrial diameter and the diameter of the aortic root, which was 2.02, indicating a left atrial dilation in this group. The echocardiographic study showed that the therapeutic vaccine with the pBCSSP4 recombinant plasmid had a better protective effect since the cardiac function was very similar to that of the healthy control group, while the pBCSP treatment showed structural and functional damage.

### 3.8. Immunotherapy with the pBCSSP4 Recombinant Plasmid Prevented Cardiomegaly

**Figure 5:**
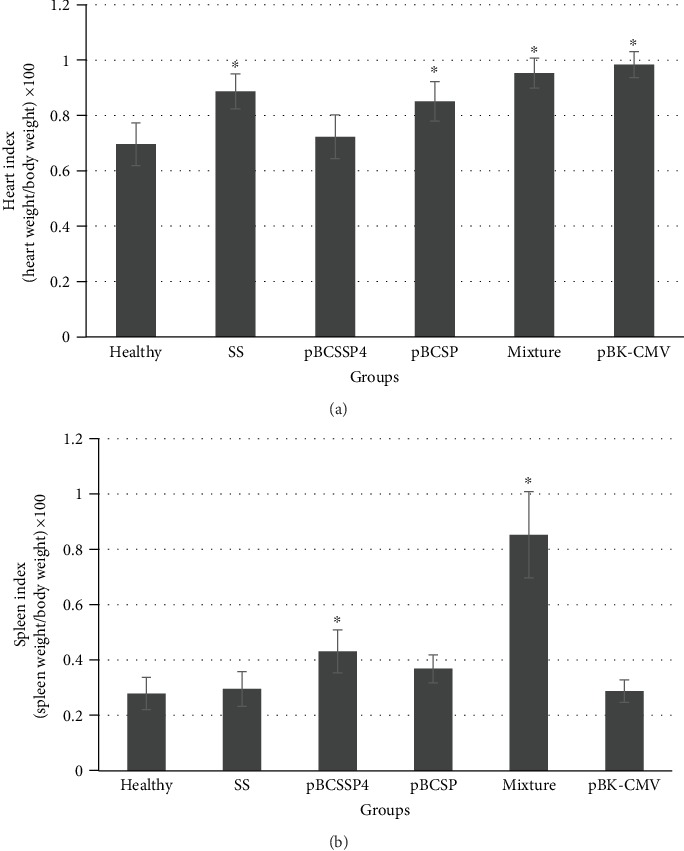
Cardiomegaly and splenomegaly during the chronic stage of infection with H8 *T. cruzi* strain in Beagle dogs treated with DNA vaccines. The enlargement of organs was calculated by the mean heart (a) and spleen (b) indices (±S.D.). Differences were considered significant at ^∗^*P* ≤ 0.05 by the Kruskal-Wallis test among the healthy group versus the SS *mock*-treated, pBCSSP4, pBCSP, mixture, and pBK-CMV groups.

After euthanasia, the heart, spleen, and lymph node indices were calculated to determine whether cardiomegaly, splenomegaly, and lymphadenopathy had occurred, respectively. The H8 *T. cruzi* strain produced cardiomegaly in all infected dogs; however, the treatment with the pBCSSP4 recombinant plasmid prevented cardiomegaly in the chronic stage of Chagas disease by showing a similar heart index than the healthy group (Figure 5(a)). A significant splenomegaly was registered in the pBCSSP4 and mixture groups in the chronic phase of Chagas disease (Figure 5(b)). Neither immunotherapy nor chronic infection caused lymph node enlargement (data not shown).

### 3.9. Both Recombinant Plasmids Separately Showed Moderate Control of the *T. cruzi* Infection by Reducing Cardiac Inflammatory Infiltrates

**Figure 6:**
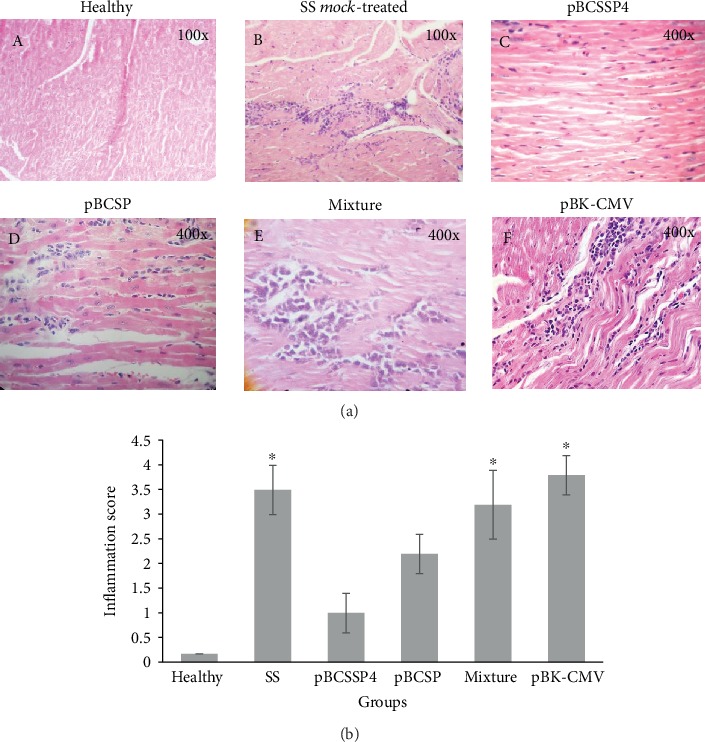
Histological ventricular myocardium findings of *T. cruzi-*infected dogs treated with DNA vaccines. (a) Representative micrographs of the heart tissue from all groups are shown. (A) Transverse section of the LV myocardium showing healthy tissue. (B) LV myocardial cross section of the SS *mock*-treated infected control group showing severe multifocal coalescent lymphoplasmacytic myocarditis (score: 4). (C) Longitudinal section of the LV myocardium of the pBCSSP4 plasmid-treated group showing mild multifocal interstitial lymphoplasmacytic myocarditis (score: 2). (D) Longitudinal section of the LV myocardium of the pBCSP plasmid-treated group showing mild multifocal interstitial lymphoplasmacytic myocarditis (score: 2) and moderate degeneration of muscle fibers. (E) RV myocardial longitudinal section of the plasmid mixture-treated group showing lymphocytic moderate multifocal coalescent myocarditis (score: 3). (F) Longitudinal section of the LV myocardium of the empty vector plasmid-treated group showing moderate to severe multifocal coalescent myocarditis (score: 4). Hematoxylin and eosin staining. (b) Inflammatory lesion (inflammatory cell infiltrates) scores. Data are expressed as the mean ± S.D., and differences were considered significant when ^∗^*P* ≤ 0.05 by the Kruskal-Wallis test for the healthy group versus the SS *mock*-treated, pBCSSP4, pBCSP, mixture, and pBK-CMV groups.

No evidence of amastigote nests, fibrosis, or edema was observed in any of the analyzed tissue sections. The healthy control group did not show histological myocardial abnormalities (Figure 6(a)). Eosinophilic lymphoplasmacytic interstitial ventricular myocarditis was observed in all infected groups at varying severity, with the SS *mock*-treated group showing severe multifocal coalescent inflammation (score: 3.5 ± 0.5) (Figures 6(a, B) and 6(b)), with both the pBCSSP4 (Figure 6(a, C)) and pBCSP (Figure 6 (a, D)) groups showing mild multifocal myocarditis (scores: 1 ± 0.4 and 2.2 ± 0.4, respectively (Figure 6(b)), and with the pBCSP group also showing moderate myofibrillar degeneration. The group treated with the mixture of both plasmids (Figure 6(a, E)) and with the empty cloning vector (Figure 6 (a, F)) showed inflammation foci similar to that found in those of the SS *mock*-treated group (scores: 3.2 ± 0.7 and 3.8 ± 0.4, respectively) (Figure 6(b)). These findings suggest that the immunotherapy with both plasmids separately moderately controlled the infection and consequently reduced the inflammatory infiltrate responsible for cardiomyopathy.

## 4. Discussion

DNA vaccines provide a new alternative for both the prevention and the treatment of a variety of infectious diseases, including Chagas disease [16]. In the development of vaccines against *Trypanosoma cruzi*, it has been suggested that to effectively control the parasite, a complete and complex immune response involving lytic antibodies and cytotoxic T cells and the production of Th1 cytokines is required [38].

The control of parasitism of *T. cruzi* depends on both innate and acquired immune responses, which are triggered during early infection and considered critical for host survival, and they both involve the participation of macrophages, natural killer (NK) cells, and T and B lymphocytes and the production of Th1 proinflammatory cytokines, such as IFN-gamma, TNF-alpha, and IL-12 [39].

In the present study, the therapeutic efficacy of two plasmids, pBCSSP4 and pBCSP, was evaluated with regard to the modulation of the immune response from the plasmid DNA vaccines in a canine model. The effect of the treatment relative to the infected/SS *mock*-treated dogs was analyzed to evaluate both the humoral and cellular immune responses through antibody production, cytokine production, and cell proliferation. On the other hand, the analysis of the general physical state, electrocardiographic and echocardiographic studies, and macroscopic findings during necropsy evaluated the degree of protection provided by this immunotherapy.

Immunotherapy with these plasmids could provide a survival advantage by reducing the clinical signs of infection and ameliorating the cardiac damage of Chagas disease by avoiding disease progression as seen with other immunotherapeutic agents against various pathologies, such as allergies, herpes, cancer, viral diseases, mycosis, Chagas disease, and leishmaniosis [40–45]. For example, in dogs naturally infected with herpesvirus, early mucosal administration of liposome-TLR complexes generated a significant reduction in the clinical signs (e.g., conjunctivitis) of canine herpesvirus infection [43]. Fever and lymph node inflammation, which were the main clinical manifestations of the acute phase in Chagas disease in dogs [46–48], were ameliorated 7-15 days after immunotherapy in 25% of DNA-treated animals.

Vaccines based on transialidases and cruzipain as either DNA vaccines, recombinant proteins, or combinations as booster or in recombinant viral vectors have shown effective prophylactic and therapeutic effects against *T. cruzi* infection in mice [20, 46–50]. In the present study, we tested two plasmids that code for two different surface proteins of *T. cruzi*: TcSSP4, an amastigote-specific glycoprotein, and TcSP, a protein of the transialidase family present in all parasitic stages [51]. Other authors found that the production of IL-6 in endothelial cells and the increased expression of mRNA of TNF-alpha and IL-1 beta in infected cardiac myocytes were induced by a transialidase, suggesting that myocytes also respond to *T. cruzi* by the production of inflammatory cytokines [39]. Consistent with studies referenced by Machado et al. [39], our pBCSP plasmid stimulated the production of IL-1 alpha and IFN-gamma better than pBCSSP4, the mixture of both plasmids, and the infection itself. It would be very convenient to correlate the cardiomyocyte integrity of vaccinated and infected dogs with their levels of these cytokines because cardiomegaly, a characteristic of Chagas disease, is attributable to local inflammatory processes related to the activation of nuclear factor kappa B (NF-kappa B) induced by Toll-like receptor 2 (TLR2) and IL-1 local production [52].

In our study, immunotherapy with the empty vector also induced similar levels of IL-12 relative to those produced by stimulation with *T. cruzi* infection and was also able to increase the levels of IFN-gamma and TNF-alpha and obtained a nonspecific response to the *T. cruzi* antigens that can be attributed to the immunomodulatory effect of the plasmid DNA as previously reported [53]. This finding is consistent with the study reported by Duan et al. [54], who immunized mice with the recombinant Sendai virus that expresses the ASP2 or UASP2 antigens of *T. cruzi* showing a protective response attributable to CD8+ T cells against *T. cruzi* infection, thus confirming the adjuvant effect of the viral vector on the activation of these cells.

CD4+ T cells play an important role in fighting pathogens via the secretion of IFN-gamma, which increases the production of nitric oxide, a substance that is toxic to intracellular parasites, and via the expression of major histocompatibility complex (MHC) class I molecules in infected cells, which allows for easier recognition by CD8+ T cells. CD8+ T cells also contribute to the elimination of intracellular pathogens by exhibiting cytotoxic activity against infected cell as well as by producing IFN-gamma after recognition of antigenic epitopes presented in combination with MHC class I molecules [54]. In this study, we found that treatment with both recombinant plasmids separately induced the expression of IFN-gamma in the acute stage of the disease, which is consistent with the findings reported by Zapata-Estrella et al. [55], who observed that immunotherapy with a plasmid encoding the TSA-1 antigen stimulated the immune system of infected mice and particularly activated IFN-gamma-producing CD4+ and CD8+ T cells in the acute and chronic phases of the experimental disease, suggesting a reorientation of the nonprotective immune response to a protective response throughout the treatment. Our recombinant plasmids used in the present study were also used in a prophylactic vaccination scheme for Chagas disease in canine models in previous studies [24], where the results indicated that vaccination with pBCSSP4 significantly increased the IFN-gamma and IL-10 levels at 9 months postinfection. In the present study, a therapeutic effect was evaluated with visible results at 45 days postinfection in the elevation of some evaluated cytokines, demonstrating that immunotherapeutic vaccines are able to redirect the immune response of infected hosts, which is consistent with Autran et al. [56]. This elevation of IFN-gamma after the second treatment when plasmids pBCSP, pBCSSP4, and the empty vector were used and the increase of more than 10 times when immunotherapy was performed with the plasmid encoding the TcSP protein after the third treatment are partially consistent with Duan et al. [54], who showed that the main target cell of their vaccination strategy was infected cells since the best immune response vaccine antigen was the ASP2 protein, which is expressed exclusively on amastigotes, an intracellular proliferative parasitic form, and expressed at higher levels compared with that in trypomastigotes, which are found in the bloodstream. The two antigens used in our study are expressed in the intracellular form of amastigotes.

Gupta and Garg used the multicomponent DNA vaccine TcVac2 and found that it stimulated a substantial response of CD8+ T cells associated with type 1 cytokines (IFN-gamma and TNF-alpha) that together resulted in acute parasitic load control. During the chronic stage, splenic activation of CD8+ T cells and these same cytokines decreased, with a predominance of IL-4/IL-10 in vaccinated mice. The role of Th1 cytokines in the immune control of *T. cruzi* has been addressed by these authors, who showed that overproduction of type 2 cytokines or blockage of type 1 cytokine production correlates with increased susceptibility to *T. cruzi* infection [57]. In our study, the concentrations of IL-4 or IL-10 were not determined; thus, we could not determine whether a certain level of susceptibility to *T. cruzi* infection could be established in infected and treated dogs due to polarization towards a certain type of immune response. Such susceptibility was reported by other studies on the development of vaccines against the parasite of the genus *Leishmania*, where the Th1/Th2 paradigm was further studied with *L. major* in mice, and the results indicated that the activation of Th1 cells producing IFN-gamma could lead to protection while Th2 cells producing IL-4 could lead to susceptibility [58].

IL-12 production was stimulated both by the infection alone and by the second injection of pBCSP plasmid as immunotherapy in infected dogs, which indicates that this plasmid is a good candidate for prophylactic and therapeutic vaccination. These effects are consistent with that described by others, who affirmed that the persistence of the *Leishmania* parasite, another trypanosomatid protozoan, and the continuous production of IL-12 are important factors for the maintenance of memory cells and long-term protective immunity [59, 60]. In contrast, immunotherapy with the plasmid pBCSSP4 induced a decrease of this cytokine in the acute stage of the infection, which is consistent with the findings reported by Ramos-Ligonio et al., who saw very low systemic and local (spleen) levels of IL-12 in immunized and infected mice when they used a recombinant SSP4 protein [61].

The elevated levels of IL-1 alpha and IL-6 produced by immunotherapy with the plasmid pBCSSP4 during the early acute stage of infection are also in accordance with Ramos-Ligonio et al., who demonstrated that the *T. cruzi* recombinant SSP4 protein is a humoral and cellular immune response modulator capable of inducing high levels of the IgG1, IgG2a, and IgG2b isotypes, the expression of inducible nitric oxide synthase, and the production of nitric oxide by macrophages as well as mRNA expression for the IL-1 alpha, IL-6, IL-12, IFN-gamma, and TNF-alpha in control infected mice and IL-10 in immunized/infected mice [61]. The results in the present study also agree with other previous reports [32], where high levels of IL-6 and TNF-alpha at 3 h and 12 h postimmunization in the sera of mice vaccinated with adjuvant with the recombinant SSP4 protein, with the empty vector, and with the pBCSSP4 were detected, suggesting that animals immunized with this gene are able to develop a Th1 response.

Gao and Pereira reported that *T. cruzi* infection in animal models and humans produces a high level of IL-6 in serum and tissue, and it is induced during the ascending phase of parasitemia in the acute stage of Chagas disease [62]. In our study, this cytokine increased with the different treatments during the early phase of the acute infection and was four times higher at the end of the acute stage (after the third treatment) with the pBCSSP4 plasmid immunotherapy. Conversely, IL-6 levels decreased with the pBCSP plasmid. IL-6 is related to B cell proliferation; however, IgG levels did not show differential production among the experimental groups because B lymphocyte proliferation occurred by another route different from that induced by IL-6, such as by IL-4, IL-5, and IL-7 [63].

Transforming growth factor beta and IL-6 are cytokines that differ significantly between cardiac patients with different stages of chagasic heart disease progression. IL-6 is a key inflammatory factor whose secretion is activated by the C-reactive protein and has been implicated in the pathogenesis and clinical evolution of cardiovascular diseases. In patients with heart failure, high serum IL-6 concentrations have been detected, which correlates with left ventricular dysfunction severity [64]. In the same way, the increased IL-6 expression by cardiac tissue has been associated with the progression of heart failure [62, 64]. Other cytokines (TNF-alpha, IL-4, IL-17, IFN-gamma, CCL2, and IL-10) have shown differences between severe chagasic heart disease and the undetermined stage but not between the different stages of chagasic heart disease progression [64]. In this regard, a difference in IL-6 and IFN-gamma production patterns was seen when immunotherapy with the different plasmid DNA was performed, which could indicate different stages of progression of heart disease as demonstrated by electrocardiography and echocardiography as well as by macro- and microscopic findings from cardiac tissue at the time of euthanasia.

Compared to the main findings on EKG reported in the chronic phase of canine Chagas disease, such as right bundle branch block, left fascicular block, ventricular premature complex, ST-T segment changes, abnormal Q waves, low-voltage QRS complex, and electric axis deviation [9, 65, 66], it is possible to assert that immunotherapy does not prevent the presentation of these alterations since most of them were found in some individuals of the experimental groups; however, the reduction in the number of abnormalities and the number of individuals treated with the pBCSSP4 and pBCSP plasmids separately was evident. The alterations found in the EKG are consistent with those reported in other studies, where the most common findings in both dogs and humans are RBBB associated with left anterior-superior fascicular block followed by various degrees of atrioventricular block [67, 68]. Respiratory sinus arrhythmia was the predominant rhythm during the assay period [68]. Sudden death was recorded in an individual who did not receive immunotherapy with recombinant plasmids which presented arrhythmia in combination with other alterations. This result is consistent with Quijano-Hernandez et al., who reported severe life-threatening cardiac arrhythmias in chagasic dogs [69].

The cardiac anatomophysiology results evaluated by echocardiography indicated that hypertrophic cardiomyopathy, characterized by the posterior wall and septum thickness [70], was the only alteration that pBCSSP4 immunotherapy was not able to prevent; however, the treatment was able to prevent other abnormalities that compromise not only the anatomy of the heart but also its functionality. Despite the presence of hypertrophic cardiomyopathy, fibrosis in myocardial tissue was not observed when performing the histological analysis, which is characteristic of many chronic diseases, including Chagas disease [70]. There were differences in the diastolic and systolic diameters when comparing the infected/SS *mock*-treated group and healthy group; and these results are not consistent with similar measurements performed by others who used young mongrel dogs infected with VL-10 strain of *T. cruzi* and did not find differences in the end-diastolic and end-systolic volumes when comparing infected and noninfected dogs [71]. On the other hand, diastolic and systolic diameters were in accordance with our previous study, in which the use of these recombinant plasmids used as a prophylactic treatment also had a protective effect based on similar parameter values found in both vaccinated and healthy control dogs with the exception of the empty vector (unpublished data).

A marked difference in the clinical and pathological conditions in the dogs infected with different *T. cruzi* strains has been described for decades. Cardiomegaly is the result of heart dysfunction in the chronic phase of Chagas disease [37, 72]. In this study, it was demonstrated that the pBCSSP4 plasmid used as therapeutic vaccine was able to prevent cardiomegaly in Beagle dogs infected with the H8 strain, and all experimental dogs that were not treated with this recombinant plasmid had a higher heart rate than the infected/SS *mock*-treated group, indicating the development of cardiomegaly. This pathological condition is in accordance with Guedes et al., who found that 20% of Beagle dogs infected with the Berenice-78 strain showed cardiomegaly, right ventricle flaccidity, inflammation, and fibrosis while 80% of the animals infected with the Y strain presented these alterations [37].

All animals showed normal popliteal node size and persistence of splenomegaly in the chronic phase of Chagas disease. These findings agree with the results reported by Guedes et al., who found similar lymphadenopathy and splenomegaly despite using different *T. cruzi* strains [73].

In this study, the therapeutic DNA vaccines carrying *T. cruzi* genes reduced multifocal myocarditis in a canine model of Chagas disease compared to unvaccinated dogs. These results are comparable to those reported in various immunoprotection studies using murine models. Using immunotherapy with the recombinant Tc24 protein reduced cardiac fibrosis by 50% [74]. In a study using a DNA vaccine containing the cruzipain gene and a plasmid encoding the granulocyte-macrophage colony-stimulating factor with *T. cruzi*-infected mice, the effects on cardiac tissue included reduced inflammatory lymphocytic foci in muscle tissue, minimal perivascular infiltrate, scarcely infiltrated and dystrophic calcifications, and absence of interfiber infiltration [75]. In another study with chagasic mice using DNA immunization with the TcG2/TcG4 glutathione peroxidase genes and recombinant proteins as reinforcement, a relative decrease in the levels of inflammatory infiltrate in the myocardium (score: 0–2, average: 0.75) was reported [76]. All these examples are in accordance with the results obtained in the present study. It has been reported that chronic Chagas cardiomyopathy is characterized by inflammatory infiltrate and extensive reactive fibrosis [77]; therefore, the results of this study are encouraging and show that immunotherapy with recombinant DNA vaccines is moderately effective since it minimizes cardiac histological damage.

## 5. Conclusions

The studied therapeutic DNA vaccines were moderately effective in preventing cardiac complications associated with chronic chagasic cardiomyopathy or in delaying their occurrence as indicated by electrocardiography. *TcSSP4* and *TcSP* genes might be candidates for future study because they may represent a new therapeutic tool against Chagas disease, which has garnered considerable interest by researchers and physicians from several countries, not only in Latin America. Immunotherapy with *T. cruzi* genes limited the severity of heart damage in experimental chagasic dogs as evaluated by electrocardiography and macroscopic findings at necropsy.

The H8 strain of *T. cruzi* used in the experimental canine infection produced splenomegaly and cardiomegaly; however, treatment with both the *TcSP* and *TcSSP4* genes prevented splenic damage but not cardiac damage during chronic Chagas disease.

In addition, treatment with the pBCSSP4 plasmid had a partial protective effect in preventing cardiomegaly and microscopic damage in cardiac tissue since dogs in this group showed cardiac indexes similar to those of the control healthy dogs and microscopic lesions covered only subepicardial tissue. All these results support the promising novel therapeutic application of DNA using the *TcSSP4* and *TcSP* genes against Chagas disease.

## Figures and Tables

**Table 1:** Study design for dogs experimentally infected with *T. cruzi* and treated with DNA vaccine containing the genes encoding a *trans*-sialidase protein (pBCSP), an amastigote-specific glycoprotein (pBCSSP4), or both as a mixture.

Group^∗^	Group description (*n*)	Plasmid used as immunotherapy
Healthy	Control noninfected/nontreated (*n* = 5)	None
Infected/SS *mock*-treated	Positive control of infection with saline solution *mock*-treated (*n* = 5)	None
pBCSSP4	Infected and pBCSSP4 plasmid-treated (*n* = 5)	Construct derived from the pBK-CMV vector with *T. cruzi* amastigote-specific glycoprotein *TcSSP4* gene
pBCSP	Infected and pBCSP plasmid-treated (*n* = 5)	Construct derived from the pBK-CMV vector with *T. cruzi trans*-sialidase *TcSP* gene
Mixture	Infected and treated with both the pBCSSP4 and pBCSP plasmids (*n* = 5)	pBCSSP4 and pBCSP plasmids carrying both genes
pBK-CMV	Infected and empty cloning vector plasmid-treated (*n* = 5)	Empty vector control of the plasmid DNA

^∗^The pBCSSP4, pBCSP, mixture, and pBK-CMV groups were treated thrice at 15-day intervals 15 days after the infection; SS was administered under this same scheme in the infected/SS *mock*-treated group.

**Table 2:** Abnormal electrocardiographic features in dogs experimentally infected with *T. cruzi* and treated with the DNA vaccine at 3, 6, and 12 months postinfection (mpi).

Group^∗^	Suggested pathological conditions by EKG recordings [78, 79]	Affected dogs (dogs/*n*) at 3 mpi	Affected dogs (dogs/*n*) at 6 mpi	Affected dogs (dogs/*n*) at 12 mpi
Inf/SS *mock*-treated	AV block+infarction	20% (1/5)		
	Ischemia	20% (1/5)		
	MEAD to the left+LBBB+infarction+ventricle enlargement	20% (1/5)		
	MEAD to the right+RBBB+infarction+ventricle enlargement		60% (3/5)	
	Arrhythmia+MEAD to the left+ventricle enlargement			20% (1/5)
	Ischemia+MEAD to the right+ventricle enlargement			60% (3/5)

pBCSSP4	AV block		20% (1/5)	
	LBBB	20% (1/5)		
	MEAD to the right+RBBB+infarction	20% (1/5)		
	MEAD to the left+LBBB+AV block	20% (1/5)		
	LBBB+infarction+ischemia	40% (2/5)	20% (1/5)	
	RBBB+ischemia			40% (2/5)

pBCSP	AV block	20% (1/5)		
	Infarction	20% (1/5)		
	Infarction+ischemia	20% (1/5)	20% (1/5)	
	Arrhythmia+AV block			20% (1/5)

Mixture	AV block+infarction+RBBB	20% (1/5)		
	Ischemia	20% (1/5)	20% (1/5)	
	Arrhythmia+ischemia		20% (1/5)	
	AV block+ischemia		20% (1/5)	20% (1/5)

pBK-CMV	Arrhythmia+ischemia		20% (1/5)	
	MEAD to the left+ventricle enlargement	40% (2/5)		
	MEAD to the left+LBBB+infarction+ventricle enlargement	20% (1/5)		
	Arrhythmia+MEAD to the right+ventricle enlargement+ischemia		20% (1/5)	
	MEAD to the right+RBBB+ventricle enlargement+infarction+ischemia		40% (2/5)	
	Ventricle enlargement			20% (1/5)
	Arrhythmia+MEAD to the left+ventricle enlargement			20% (1/5)
	Arrhythmia+ventricle enlargement+ischemia+MIMI			20% (1/5)

^∗^Group descriptions are shown in Table 1. mpi = months postinfection; Inf = infected; AV = atrioventricular; LBBB = left bundle branch block; RBBB = right bundle branch block; MEAD = mean electrical axis deviation; MIMI = microscopic intramural myocardial infarction.

**Table 3:** Cardiovascular parameters based on echocardiography in dogs experimentally infected with *T. cruzi* and treated with the DNA vaccine containing genes encoding a *trans*-sialidase protein (pBCSP), an amastigote-specific glycoprotein (pBCSSP4), or both as a mixture.

Group^∗^	Left ventricular (LV) diastolic diameter (mm)	Left ventricular (LV) systolic diameter (mm)	Fractional shortening (FS) (%)	Left ventricular ejection fraction (LVEF) (%)	Posterior wall (mm)	Septum (mm)	Left atrium (LA) diameter/aorta root (AR) diameter ratio
Healthy	30.25 ± 3.89	18.46 ± 2.07	42.12 ± 4.98	53.20 ± 3.83	5.86 ± 0.21	6.35 ± 0.21	1.73 ± 0.095
Inf/SS *mock*-treated	28.33 ± 3.32^∗^	15.60 ± 1.57^∗^	44.75 ± 3.97	50.31 ± 6.31	6.81 ± 0.40^∗^	8.00 ± 1.19^∗^	2.23 ± 0.19^∗^
pBCSSP4	30.63 ± 4.88	20.00 ± 4.55	38.33 ± 5.90	50.15 ± 8.99	6.85 ± 0.84^∗^	7.00 ± 0.94	1.92 ± 0.28
pBCSP	32.45 ± 5.89	20.00 ± 2.92	29.24 ± 3.36	43.25 ± 3.78^∗^	5.65 ± 0.45	6.30 ± 0.78	2.02 ± 0.18^∗^
Mixture	35.60 ± 2.74	23.85 ± 4.09	38.37 ± 9.36	49.75 ± 8.66	6.34 ± 0.92	7.08 ± 0.50	1.79 ± 0.40
pBK-CMV	30.18 ± 3.66	17.85 ± 1.57	38.05 ± 6.70	50.50 ± 6.10	6.40 ± 0.49	6.74 ± 0.71	1.78 ± 0.33

^∗^Group descriptions are shown in Table 1. Inf = infected. All data are expressed as the means and standard deviations. ^∗^Significant difference (*P* ≤ 0.05) between the treatment groups and the healthy dogs and/or with reference values [80].

## Data Availability

The datasets generated and/or analyzed during the current study are available from the corresponding author on reasonable request.
